# The effect of peer support in diabetes self-management education on glycemic control in patients with type 2 diabetes: a systematic review and meta-analysis

**DOI:** 10.4178/epih.e2021090

**Published:** 2021-10-22

**Authors:** Akhmad Azmiardi, Bhisma Murti, Ratih Puspita Febrinasari, Didik Gunawan Tamtomo

**Affiliations:** 1 Doctoral Program on Public Health, Universitas Sebelas Maret, Surakarta, Indonesia; 2 Department of Public Health, Universitas Veteran Bangun Nusantara, Sukoharjo, Indonesia; 3 Department of Pharmacology, Faculty of Medicine, Universitas Sebelas Maret, Surakarta, Indonesia

**Keywords:** Peer support, Randomized controlled trial, Type 2 diabetes, Meta-analysis

## Abstract

**OBJECTIVES:**

Diabetes self-management education (DSME) programs are a strategy to maintain healthy behaviors. Nevertheless, limited evidence has been reported from systematic evaluations of the effects of DSME integrated with peer support on glycemic control. This study aimed to review the effectiveness of DSME interventions integrated with peer support on glycemic control in patients with type 2 diabetes.

**METHODS:**

A systematic search was carried out in electronic databases, including PubMed, Cochrane Library, ProQuest, SpringerLink, ScienceDirect, Scopus, and Google Scholar, for English-language articles published from 2005 until 2020. The effect size was estimated as the standard mean difference (SMD). The Cochrane Collaboration’s Risk of Bias tool was employed to assess the risk of bias.

**RESULTS:**

Twelve studies were included in this study. DSME integrated with peer support effectively reduced glycated hemoglobin A1c (HbA1c) levels, with a statistically significant effect (SMD, -0.41; 95% confidence interval [CI], -0.69 to -0.13; p<0.001). Programs with a sample size <100 (SMD, -0.45; 95% CI, -0.79 to -0.11; p=0.009), duration of intervention ≤6 months (SMD, -0.52; 95% CI, -0.96 to -0.07; p=0.020), baseline HbA1c <8.5% (SMD, -0.42; 95% CI, -0.77 to -0.07; p=0.020), delivery by group (SMD, -0.28; 95% CI, -0.51 to -0.06; p=0.010), and high frequency of contact (SMD, -0.29; 95% CI, -0.48 to -0.10; p=0.003) had statistically significant effects on reducing HbA1c levels in patients with type 2 diabetes.

**CONCLUSIONS:**

DSME integrated with peer support effectively enhances glycemic control in patients with type 2 diabetes. Programs with smaller participants groups, shorter interventions, weekly meetings, and closer group sessions improved glycemic control in patients with type 2 diabetes.

## INTRODUCTION

The prevalence of diabetes has dramatically grown in all regions and countries worldwide, with increases of 2-4% per year. The global prevalence of diabetes in 2019 was estimated to be 9.3% (463 million individuals), and is anticipated to grow to 10.2% (578 million) by 2030 and 10.9% (700 million) by 2045. Type 2 diabetes accounts for more than 90% of patients with diabetes [1]; in addition to its high prevalence and incidence, the serious complications and increased mortality associated with type 2 diabetes have made it a major healthcare problem in all nations [2].

Type 2 diabetes is a chronic disease, the major characteristic of which is hyperglycemia [3]. Severe hyperglycemia in diabetes leads to chronic and acute complications [4]. Acute complications include diabetic ketoacidosis and hypoglycemia, while chronic complications can be microvascular (retinopathy, nephropathy, neuropathy) or macrovascular (peripheral arterial disease, stroke, ischemic heart disease) [3].

Patients with type 2 diabetes need complex care to control acute complications and reduce their risk of long-term complications. Patients with type 2 diabetes require not only ongoing medical care, but also diabetes self-management, which patients must do themselves. Effective glycemic control is a vital component of the management of patients with type 2 diabetes. A healthy lifestyle, appropriate diet, and medication adherence are essential factors for good glycemic control [5].

The American Diabetes Association has recommended glycated hemoglobin A1c (HbA1c) as the established standard measurement for assessing glycemic control in individuals with diabetes. Following the guidelines, an HbA1c level of around 7% indicates good glycemic control [2]. HbA1c reflects glycemic control over the past 2 months or 3 months, and it is broadly used as a reference for the prognosis, diagnosis, and therapy in patients with diabetes mellitus (DM) [6].

However, many patients with diabetes fail to reach glycemic control targets due to the complex nature of type 2 diabetes self-management. Hence, patients with diabetes need self-management education to assist them in comprehending and dealing with the disease. Diabetes education about health behaviors, including diet, medication adherence, blood glucose self-monitoring, and physical activity, is essential for successful diabetes management [7].

Diabetes self-management education (DSME) is a strategy used to optimize glycemic control by educating people about self-management and helping people to maintain healthy behaviors. DSME encourages patients to acquire the information, abilities, and capacities necessary for diabetes self-care [8]. DSME programs are also effective for preventing the complications of diabetes and enhancing health outcomes in patients with diabetes. Previous systematic reviews have reported that DSME programs were effective for diabetes outcomes, including glycemic control, knowledge of diabetes, self-management skills, and self-efficacy [9,10].

DSME is vital for diabetes self-management, but sustaining appropriate behaviors is also required to maintain patient self-management in an ongoing program. In this regard, peer support may be a critical way to provide successful diabetes self-management [11,12]. Peer support in diabetes self-management allows patients to engage in mutual knowledge-sharing, collaborative problem-solving, and emotional support for the stresses of dealing with type 2 diabetes.

However, although the benefits of DSME have been discussed [13,14], to the best of the authors’ knowledge, few meta-analyses have explicitly investigated peer support in the literature on DSME. Hence, this meta-analysis aimed to investigate DSME interventions integrated with and focused on peer support as an essential component of diabetes self-management and to examine the effect of DSME interventions integrated with peer support on enhancing glycemic control in patients with type 2 diabetes.

## MATERIALS AND METHODS

A meta-analysis was conducted of randomized controlled trials (RCTs) comparing peer support integrated with DSME with usual care or less intense self-management interventions. This meta-analysis was reported in accordance with the PRISMA (Preferred Reporting Items for Systematic Reviews and Meta-Analyses) guideline [15].

### Search strategy

A comprehensive search was conducted to find relevant English-language articles from electronic databases and the gray literature published between 2005 and 2020. Electronic databases including PubMed, Cochrane Library, ProQuest, SpringerLink, ScienceDirect, Scopus, and Google Scholar were utilized to search for relevant articles. Literature searches were carried out to identify studies investigating the effects of peer support integrated with a DSME program on enhancing glycemic control in patients with type 2 DM. An initial search was performed based on the PICO (participants, comparison, intervention, and outcomes) framework and key terms. The following key terms were used: “type 2 diabetes”[MeSH] OR “type 2 diabetes mellitus”[tiab] AND “diabetes self-management education”[tw] OR “DSME”[tw] AND “peer”[tiab] AND “peer support”[tiab] AND “peer group”[tw] AND “glycosylated hemoglobin”[MeSH] OR “HbA1c”[tw] AND “glycemic control”[tw]. In addition, publications from non-profit organizations, such as the Centers for Disease Control and Prevention, World Health Organization, and American Diabetes Association, were employed to search the gray literature.

### Inclusion and exclusion criteria

In this study, the inclusion criteria were RCTs including individuals with type 2 diabetes that used DSME integrated with peer support as an intervention program. Studies were included if they were non-RCTs, did not use DSME as intervention, did not include a peer support component, had an inappropriate population, or did not measure HbA1c as the outcome.

### Study selection

A screening process was independently conducted by 2 authors (BM and DGT). In the first stage, reviewers independently extracted information from potentially relevant titles and abstracts. The screened studies were then included in the second stage for a full-text review. Again, independently, the 2 authors read and evaluated the full-text articles based on predefined exclusion and inclusion criteria. Finally, the 2 authors compared the results, and any differences were resolved by reaching a consensus. Through this process, articles qualifying for the meta-analysis were included.

### Extraction of data

Two authors independently (AA and BM) extracted data from the included articles into a structured table. The extracted data consisted of the first author, year of publication, study design, settings, country, duration of diabetes, mean participant age, sample size, and HbA1c level at baseline and end of the study. Additionally, information was recorded on the intervention characteristics, including the type of enrollment criteria for peers, peer support, training for peers, frequency of contact, mode of delivery, number of contact hours per session, duration of the intervention, and definitions of the intervention and control groups.

### Bias and quality assessment

This study used the Cochrane Collaboration’s Risk of Bias tool to evaluate the risk of bias in the included studies by 2 authors independently (BM and DGT). The risk of bias was examined using the Cochrane Collaboration’s Risk of Bias tool in terms of the following criteria: allocation concealment (selection bias), random sequence generation (selection bias), blinding participants and personnel (performance bias), incomplete outcome data (attrition bias), blinding of outcome assessment (detection bias), selective reporting of outcomes (reporting bias), and other biases [16]. The risk of bias for each domain was rated as high, low, or unclear.

### Statistical analysis

By utilizing Review Manager (RevMan) version 5.3 (Cochrane Collaboration, London, UK) and Stata version 16 (StataCorp., College Station, TX, USA), statistical analyses were performed to investigate the effects of interventions on glycemic control. The extracted data, including the number of participants, mean, and standard deviation, were entered into RevMan. The effect size was calculated as the standardized mean difference (SMD) with a confidence interval (CI) of 95%, and a 2-sided p-value less than 0.05 signifying a statistically significant difference between groups. The pooled SMD was utilized to estimate the effect of peer support integrated with DSME on glycemic control in patients with type 2 diabetes.

The heterogeneity between studies was measured statistically by using the intuitive index (I^2^), which reflects the total variation across studies (described as a percentage) because of heterogeneity instead of sampling error [17]. An I^2^ value of more than 50% indicates substantial heterogeneity [18]. Random-effect analysis models are used if heterogeneity is detected by an I^2^ value more than 50% [19]. Publication bias was assessed by the Begg and Egger tests [20,21], as well as the funnel plot asymmetry test. A symmetrically distributed shape of a funnel plot indicates no potential publication bias; otherwise, an asymmetrical shape of a funnel plot signifies potential publication bias [22]. Potential publication bias was identified statistically if both the Begg and Egger tests had p-values less than 0.05.

In order to provide more detailed results, the authors performed subgroup analyses on the basis of the studies’ characteristics. The following subgroups were included: (1) sample size, divided into < 100 or ≥ 100; (2) intervention duration, divided into ≤6 months or >6 months; (3) baseline HbA1c (<8.5 or ≥8.5%); (4) the type of intervention delivery, divided into the individual, group, and combinations of individual and group interventions; and (5) the frequency of contacts, divided into low (less than 1 contact per month), moderate (1 or 2 contacts per month), and high (more than 2 contacts in a month).

### Ethics statement

This article does not contain any studies with human participants performed by any of the authors.

## RESULTS

**Figure 1.**
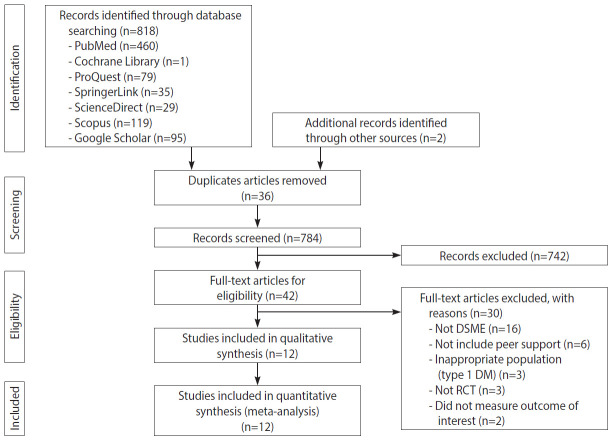
PRISMA (Preferred Report ing Items for Systematic Reviews and Meta-Analyses) flowchart. DSME, diabetes self-management education; DM, diabetes mellitus; RCT, randomized controlled trial.

In total, 820 published articles were collected from online databases, including PubMed, ProQuest, Cochrane Library, ScienceDirect, SpringerLink, and Google Scholar. All articles were published between 2005 to 2020. After deleting duplicates, 784 abstracts were obtained. After the review of abstracts, 42 articles were chosen for full-text review. Thirty articles were excluded for various reasons, such as the intervention not meeting the established criteria for DSME, not including peer support components, having an inappropriate population (e.g., type 1 DM), not having a RCT design, and not measuring the outcome of interest. Twelve articles were selected for the qualitative synthesis, and 12 articles were eligible according to the inclusion criteria [23-34]. Figure 1 presents the PRISMA flowchart of the article selection process.

### Characteristics of the included studies

The characteristics of the included studies can be found in Table 1. Data were extracted on the author, year of publication, country, study design, setting, age, sample size, HbA1c levels, type of peer support, type of delivery, frequency of contact, and duration of intervention. Five studies were carried out in the United States and 1 each in Vietnam, China, Jamaica, the Philippines, Australia, Canada, and Hong Kong. Overall, 1,896 participants were included in this study from all articles. The sample size of studies ranged from 31 to 159. The mean HbA1c levels at baseline ranged from 6.3% to 10.5%. Meanwhile, the mean HbA1c levels after the intervention fluctuated from 6.4% to 9.7%.

### Bias and quality of the included studies

Because of random sequence generation, 6 studies had a low risk of selection bias, while the risk of bias was unclear for 6 studies. Regarding concealment of allocation, 3 studies described a high risk of bias, 1 study had a low risk of bias, and 8 studies did not describe allocation concealment. The risk of performance bias because of blinding of personnel and participants was high for 4 studies and low for 3 studies, whereas 5 studies did not explain the blinding of personnel and participants. Two studies had a high risk of detection bias based on their description of the blinding of outcome assessment, 3 studies had a low risk of bias, and 7 studies did not describe this process. There was a low risk of attrition bias due to incomplete outcome data in 8 studies, 3 studies did not describe incomplete outcomes, and only 1 study described a high risk of bias. All 12 studies had a low risk of bias for selective reporting. In terms of other sources of bias, 4 studies had a high risk based on their descriptions, 7 studies did not contain a relevant description, and 1 study had a low risk of other bias (Supplementary Material 1). The overall evidence was originally evaluated as highquality. However, due to the heterogeneity of the study findings, the quality of evidence was downgraded from high to moderate.

### Intervention effects on glycemic control

**Figure 2.**
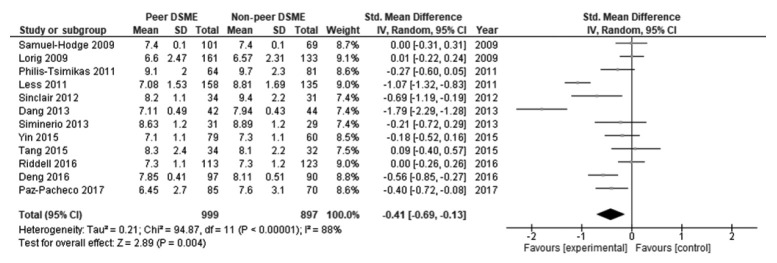
Forest plot of the overall included studies. DSME, diabetes self-management education; SD, standard deviation; CI, confidence interval.

Table 2 displays the effect size and 95% CI of the included studies. The peer support programs’ impact on patients’ HbA1c was evaluated in 12 studies. Due to significant heterogeneity, a random-effect model was employed to evaluate possible HbA1c differences between the control and intervention groups. There was a decrease in HbA1c levels in the intervention group compared to the control group. The pooled effect size (SMD) was -0.41 (95% CI, -0.69 to -0.13), and it was statistically significant (p=0.004), favoring peer support over usual care. High and statistically significant heterogeneity was found among the studies in terms of changes in HbA1c (I^2^ = 88%, p<0.001) (Figure 2). The results of the publication bias assessment showed no statistically publication bias according to the Begg test (p=0.410) and Egger test (p=0.519), and a symmetrical funnel plot indicated no risk of publication bias (Supplementary Material 2).

### Subgroup analysis based on sample size

A subgroup analysis was performed for sample size (< 100 vs. ≥ 100). The effect size was larger in studies with a sample size < 100 (SMD, -0.45; 95% CI, -0.79 to -0.11) than in those with a sample size ≥ 100 (SMD, -0.33; 95% CI, -0.87 to 0.20), and the effect size was only statistically significant in the smaller studies (p=0.009 vs. p=0.220). The heterogeneity was higher for studies with a sample size ≥ 100 (I^2^=94%) than in those with a sample size < 100 (I^2^=84%), and it was statistically significant in both groups (p<0.001 for both). No potential publication bias was detected in studies with a sample size < 100 according to the Begg test (p=0.620) and Egger test (p=0.413) or in those with a sample size ≥ 100 according to the Egger test (p=0.988) and Begg test (p=0.496).

### Subgroup analysis based on duration of intervention

A subgroup analysis was done for studies with intervention durations of ≤ 6 months and > 6 months. The effect size was larger in studies with a ≤ 6-month intervention duration (SMD, -0.52; 95% CI, -0.96 to -0.07) than in those with a > 6-month duration (SMD, -0.32; 95% CI, -0.71 to 0.08), and it was only statistically significant in the shorter group (p=0.020 vs. p=0.120). The heterogeneity was lower in the studies with a ≤ 6-month intervention duration (I^2^=89%) than in those with a > 6-month duration of the intervention (I^2^=90%), and it was statistically significant in both groups (p<0.001 in both). According to the Begg test (p=0.188) and the Egger test (p=0.108) for studies with a ≤ 6-month duration of intervention, no potential publication bias was detected. Likewise, no potential publication bias was detected for studies with a > 6-month duration of intervention according to the Begg test (p=0.573) and the Egger test (p=0.358).

### Subgroup analysis based on the baseline HbA1c level

A subgroup analysis was conducted for according to the baseline HbA1c level (< 8.5 vs. ≥ 8.5%). There was larger effect size in studies where the participants had a baseline HbA1c level < 8.5% (SMD, -0.42; 95% CI, -0.77 to -0.07) than in those where the participants had baseline HbA1c levels ≥ 8.5% (SMD, -0.36; 95% CI, -0.62 to -0.10); the effect size was statistically significant in both groups (p=0.020 and p=0.006, respectively). There was high and significant heterogeneity in studies with a baseline HbA1c level < 8.5% (I^2^=91%, p<0.001), while heterogeneity was low and non-significant in studies with a baseline HbA1c ≥ 8.5% (I^2^=11%, p=0.320). Publication bias was not detected according to the Begg test (p=0.404) or the Egger test (p=0.540) for studies with a baseline HbA1c < 8.5%. No publication bias was detected for studies with a baseline HbA1c ≥ 8.5% according to the Begg test (p=0.601) or the Egger test (p=0.692).

### Subgroup analysis based on the type of intervention delivery

A subgroup analysis was carried out based on the type of intervention delivery (group or combination of individual and group). There was no subgroup analysis for individual delivery alone because there were no programs with individual delivery alone included in the studies. There were significant differences in the HbA1c level in participants in studies with group delivery (SMD, -0.28; 95% CI, -0.51 to -0.06; p=0.010). The effect size among participants in interventions with a combined delivery was larger than in those with group delivery, but it was not statistically significant (SMD, -0.53; 95% CI, -1.07 to 0.02; p=0.060). The heterogeneity was higher in the studies with a combined delivery (I^2^=92%) than in the studies with group delivery (I^2^=70%), and it was statistically significant in both groups (p<0.001 and p=0.005, respectively). Publication bias was not identified by the Begg test (p=0.188) or the Egger test (p=0.072) for the studies with group delivery, and no potential publication bias existed according to the Begg test (p=0.851) or the Egger test (p=0.780) for studies with a combined delivery.

### Subgroup analysis based on the frequency of contact

A subgroup analysis was conducted on the basis of the frequency of contact (low, moderate, or high). Statistically significant differences existed in HbA1c levels in participants of interventions with high contact frequency (SMD, -0.29; 95% CI, -0.48 to -0.10; p=0.003). In the contrary, no statistically significant differences were found in HbA1c levels in participants of interventions with both low (SMD, -0.53; 95% CI, -1.60 to 0.54; p=0.330) and moderate contact frequency (SMD, -0.65; 95% CI, -1.71 to 0.40; p=0.220). The heterogeneity was high and statistically significant for interventions with both low (I^2^=98%; p<0.001) and moderate contact frequency (I^2^=95%; p<0.001) than for those with high contact frequency, although heterogeneity was also significant in the high contact frequency group (I^2^=52%; p=0.050). There was no potential for publication bias detected in the interventions with moderate contact frequency according to the Begg test (p=0.601) and the Egger test (p=0.494) or for those with high contact frequency according to the Begg test (p=0.652) and the Egger test (p=0.933). In the low contact frequency group, a risk for publication bias was not statistically identified by the Begg test (p=0.317), but a risk was detected by the Egger test (p<0.001). We presented forest plot of subgroup analyses in Supplementary Material 3.

## DISCUSSION

This meta-analysis investigated the effect of peer support integrated with DSME on glycemic control in patients with type 2 DM. A systematic review was conducted of 12 RCTs. Only RCTs were included in this meta-analysis study to ensure high validity of the results. The interventions consisted of peer support integrated with DSME compared to usual or standard care in patients with type 2 diabetes. This study found that peer support integrated with DSME significantly reduced HbA1c levels and improved glycemic control among participants in the intervention groups.

This meta-analysis also found a significant impact of peer support integrated with DSME on HbA1c. There was a -0.41% (95% CI, -0.69 to -0.13) reduction in HbA1c in people who received peer support integrated with DSME management treatment. The estimated 0.41% improvement in glycemic control indexed by HbA1c was modest. However, the evidence suggests that modest improvements in glycemic control are large enough to generate significant reductions in the risk of developing microvascular or macrovascular complications [35,36]. A similar improvement in glycemic control was seen in another RCT with an educational program for type 2 diabetes [37,38]. The findings of this meta-analysis align with a previous DSME meta-analysis that found a reduction in HbA1c with significant results in patients with diabetes [39]. This study has significant implications for clinical research and practice in diabetes or public health. Glycemic control, as indexed by HbA1c, provides a reliable measure of hyperglycemia. HbA1c is also a significant indicator for predicting future complications [35,40,41].

Moreover, there was significant and high heterogeneity among the included studies (I^2^=88%, p<0.001). This high heterogeneity suggests substantial inconsistency in the effects, as reflected by variation in the intervention characteristics. Due to this inconsistency, the quality of evidence was downgraded from high to moderate.

Subgroup analyses were also performed to scrutinize the effect of peer support integrated with DSME on glycemic control based on intervention characteristics, including sample size (< 100 or ≥ 100), duration of the intervention (≤ 6 or > 6 months), HbA1c at baseline (< 8.5 or ≥ 8.5%), type of intervention delivery (individual, group, or combination), and frequency of contact (low, moderate, or high).

The subgroup analysis based on sample size showed larger effects (SMD, -0.45) in small studies than in large studies, and the effect in smaller studies was statistically significant (p=0.009). This result is in line with a previous meta-analysis stating that sample size was associated with intervention duration. Smaller studies had shorter interventions, indicating that compact interventions may have a more significant effect [42].

The analysis of the intervention duration revealed a larger and significant effect in short interventions (SMD, -0.52) than in long interventions. This result is in line with prior evidence. Thus, the evidence suggests that behavioral and educational interventions with a short duration and follow-up are more effective in improving HbA1c levels, medication adherence, healthy diet, and physical activity [43,44].

The subgroup analysis results based on the baseline HbA1c value showed a significant effect. Interventions where participants’ baseline HbA1c levels were ≤ 8.5% had a larger effect, and it was statistically significant (SMD, -0.42; p=0.020), than those where the baseline HbA1c levels were≥ 8.5%. The evidence suggests that mean values of HbA1c baseline less than 9% are associated with glycemic control enhancement [45]. A higher baseline HbA1c may be correlated with a longer duration of diabetes. A shorter diabetes duration may be associated with a greater effect than a longer diabetes duration, since participants with a longer duration of diabetes may find it more difficult to change.

The analysis according to the type of intervention delivery revealed that group interventions had a statistically significant effect on HbA1c levels (SMD, -0.28; p=0.010), and this effect was higher than interventions with other types of delivery. This result aligns with a previous meta-analysis showing that group interventions were better at optimizing outcomes for diabetes [44,46].

Peer support with a high contact frequency showed a statistically significant effect in terms of glycemic control (SMD, -0.29; p=0.003), and the effect size was higher than those of interventions with a low or moderate frequency of contact. This outcome is consistent with those of prior meta-analyses suggesting that a higher frequency of contact or intensity effectively improved glycemic control [42,47]. This finding signifies that peer-support interventions with a high frequency of contact should be implemented in participants with diabetes.

### Strength

This study has several strengths. Firstly, the PRISMA method was used to conduct a meta-analysis and systematic review. Secondly, a broad search strategy was employed to collect all relevant articles. Thirdly, 2 independent reviewers conducted the review process described in this study. Fourthly, only RCTs were included in this study to ensure high validity. Fifthly, subgroup analyses based on the study characteristics were conducted to identify essential findings.

### Limitation

There are several limitations of this review. First, this study was limited to only English-language articles; therefore, publication bias may have been an issue even though it was not detected by statistical tests. Moreover, the researchers were not aware of any unpublished articles that fulfilled this study’s criteria. Third, the quality of evidence was downgraded from high to moderate quality due to the high overall heterogeneity. Furthermore, the subgroup analyses were limited to only 5 characteristics, although future research could explore more characteristics, such as the study setting, participants’ education levels and socioeconomic status, the duration of diabetes, and complications of diabetes. Finally, there was a limited number of studies on peer support with DSME programs, meaning that further research is needed for more evidence. In addition, we were unable to evaluate the quality of peer support and intervention programs in each study. Variations may have existed due to potential differences in components of the program such as peer interactions, supervision, and education.

## CONCLUSION

Peer support integrated with DSME effectively enhances glycemic control in patients with type 2 diabetes. In addition, interventions with smaller groups, shorter durations, lower HbA1c baseline levels, group sessions, and high frequencies of sessions significantly enhanced glycemic control in patients with type 2 DM.

Further clinical trials exploring peer support components integrated with DSME in different configurations are needed to obtain more substantial evidence in clinical practice both to find the most effective regimen and to determine the most cost-effective model for patients with type 2 DM. Healthcare providers should design compact intervention programs, involving smaller groups, shorter durations, weekly meetings, and closer group sessions with peer support involvement in order to implement self-management practice and improve glycemic control in patients with type 2 diabetes.

## Figures and Tables

**Table 1.** Characteristics of the included studies

Study	Country	Design	Setting	Age (yr)		Baseline sample size		End of study sample size		HbA1c at baseline, mean±SD		HbA1c after intervention, mean±SD		Type of delivery	Frequency of contact	Duration of intervention (mo)
				IG	CG	IG	CG	IG	CG	IG	CG	IG	CG			
Dang et al., 2013 [23]	Vietnam	RCT	Outpatient clinic	≥30	≥30	47	50	42	44	7.96±0.67	7.85±0.34	7.11±0.49	7.94±0.43	Combined	Moderate	6
Deng et al., 2016 [24]	China	RCT	Community	57.5	56.3	97	111	97	90	8.45±0.39	8.43±0.47	7.85±0.41	8.11±0.51	Group	High	7
Less et al., 2011 [34]	Jamaica	RCT	Health centers	56.6	58.6	159	159	158	135	7.94±2.12	8.08±1.98	7.08±1.53	8.81±1.69	Combined	Low	12
Lorig et al., 2009 [25]	USA	RCT	Community	67.7	65.4	186	159	161	133	6.70±1.48	6.74±1.38	6.60±2.47	6.57±2.31	Group	Low	6
Paz-Pacheco et al., 2017 [27]	Philippine	RCT	Health center	57.6	56.5	85	70	85	70	6.35±3.95	7.25±3.70	6.45±2.7	7.60±3.1	Group	High	6
Riddell et al., 2016 [28]	Australia	RCT	Community	61.3	60.5	138	136	113	123	7.3±1.0	7.2±1.3	7.3±1.1	7.3±1.2	Group	Moderate	12
Siminerio et al., 2013 [29]	USA	RCT	Community	64.0	60.0	36	32	31	29	8.6±2.4	8.7±1.9	8.6±1.2	8.9±1.2	Combined	Moderate	6
Sinclair et al., 2013 [30]	USA	RCT	Community	53.0	55.0	48	34	34	31	9.9±2.0	9.8±2.2	8.2±1.1	9.4±2.2	Group	High	3
Tang et al., 2015 [31]	Canada	RCT	Community	56.7	55.9	54	52	34	32	7.8±2.1	8.0±1.6	8.3±2.4	8.1±2.2	Combined	High	15
Yin et al., 2015 [32]	Hong Kong	RCT	Hospital or community-based clinic	55.6	56.5	79	60	79	60	7.1±0.3	7.1±0.5	7.1±1.1	7.3±1.1	Combined	High	6
Philis-Tsimikas et al., 2011 [33]	USA	RCT	Community	52.2	49.2	104	103	64	81	10.5±1.7	10.3±1.7	9.1±2.0	9.7±2.3	Group	High	10
Samuel-Hodge et al., 2009 [26]	USA	RCT	Churches	57.0	61.3	117	84	101	69	7.7±0.2	7.9±0.3	7.4±0.1	7.7±0.1	Combined	High	12

HbA1c, glycated hemoglobin A1c; SD, standard deviation; IG, intervention group; CG, control group; RCT, randomized controlled trial.

**Table 2.** Subgroup analyses

Variables		No. of studies	HbA1c, SMD (95% CI)	p-value	Heterogeneity		Begg test	Egger test
					I^2^ (%)	p-value	p-value	p-value
All studies		12	-0.41 (-0.69, -0.13)	0.004	88	<0.001	0.410	0.519
Sample size								
	<100	8	-0.45 (-0.79, -0.11)	0.009	84	<0.001	0.620	0.413
	≥100	4	-0.33 (-0.87, 0.20)	0.220	94	<0.001	0.496	0.988
Duration of intervention (mo)								
	≤6	6	-0.52 (-0.96, -0.07)	0.020	89	<0.001	0.188	0.108
	>6	6	-0.32 (-0.71, 0.08)	0.120	90	<0.001	0.573	0.358
Baseline HbA1c (%)								
	<8.5	9	-0.42 (-0.77, -0.07)	0.020	91	<0.001	0.404	0.540
	≥8.5	3	-0.36 (-0.62, -0.10)	0.006	11	0.320	0.601	0.692
Type of intervention delivery								
	Individual	0	-	-	-	-	-	-
	Group	6	-0.28 (-0.51, -0.06)	0.010	70	0.005	0.188	0.072
	Combined	6	-0.53 (-1.07, 0.02)	0.060	92	<0.001	0.851	0.780
Frequency of contact								
	Low	2	-0.53 (-1.60, 0.54)	0.330	98	<0.001	0.317	<0.001
	Moderate	3	-0.65 (-1.71, 0.40)	0.220	95	<0.001	0.601	0.494
	High	7	-0.29 (-0.48, -0.10)	0.003	52	0.050	0.652	0.933

SMD, standard mean difference; HbA1c, glycated hemoglobin A1c; CI, confidence interval.
