# Supplementary material for: The effect of peer support in diabetes self-management education on glycemic control in patients with type 2 diabetes: a systematic review and meta-analysis
Source: Epidemiol Health. 2021 Oct 22;43:e2021090. doi: 10.4178/epih.e2021090 (PMC8920738; doi:10.4178/epih.e2021090)
Supplement: Supplementary Material 1. [file epih-43-e2021090-suppl1.pdf]

Supplementary Material 1

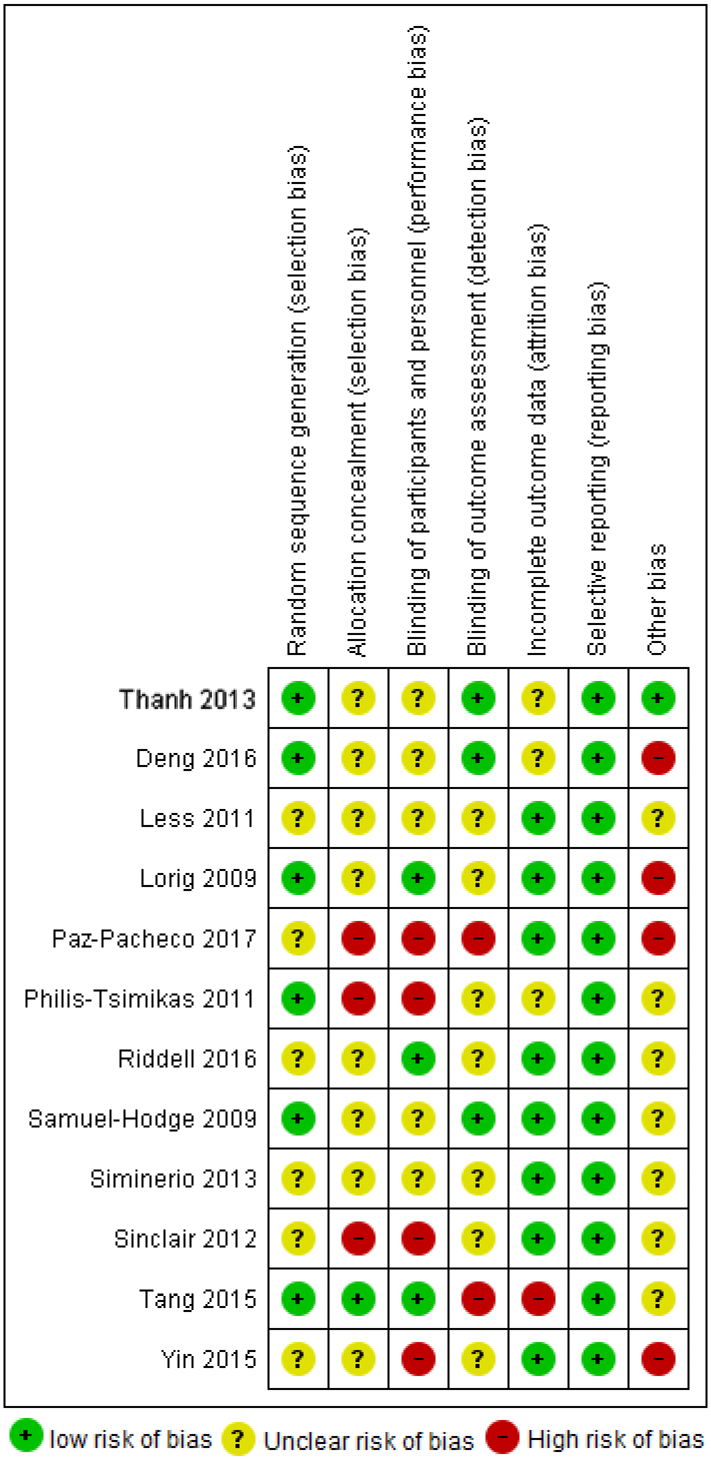

Figure 1. Risk of Bias Summary

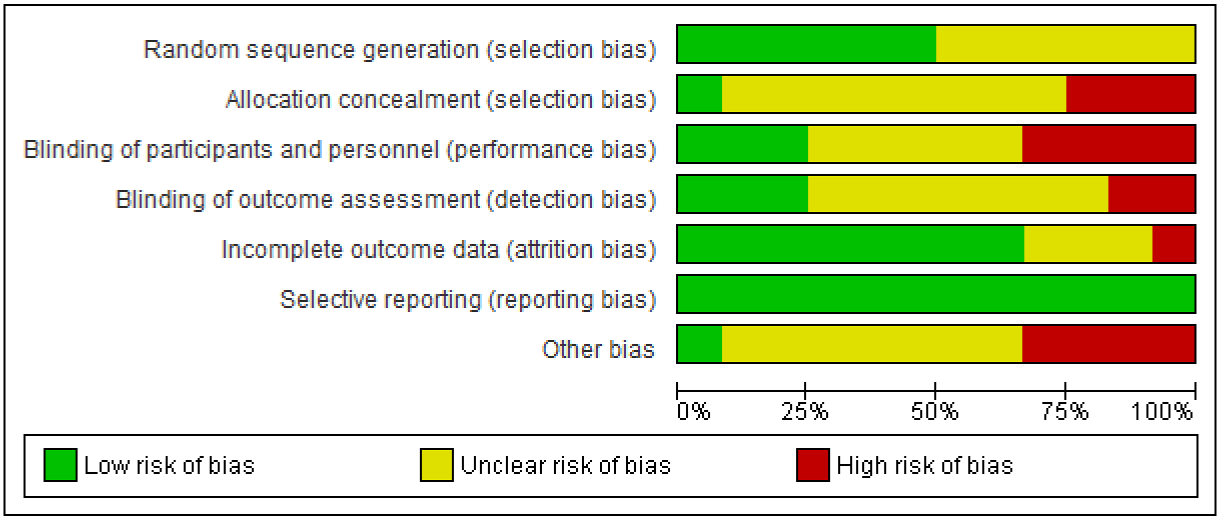

Figure 2. Risk of Bias Graph
