# Supplementary figures and images for: The effect of peer support in diabetes self-management education on glycemic control in patients with type 2 diabetes: a systematic review and meta-analysis
Source: Epidemiol Health. 2021 Oct 22;43:e2021090. doi: 10.4178/epih.e2021090 (PMC8920738; doi:10.4178/epih.e2021090)

# Supplementary Material 2

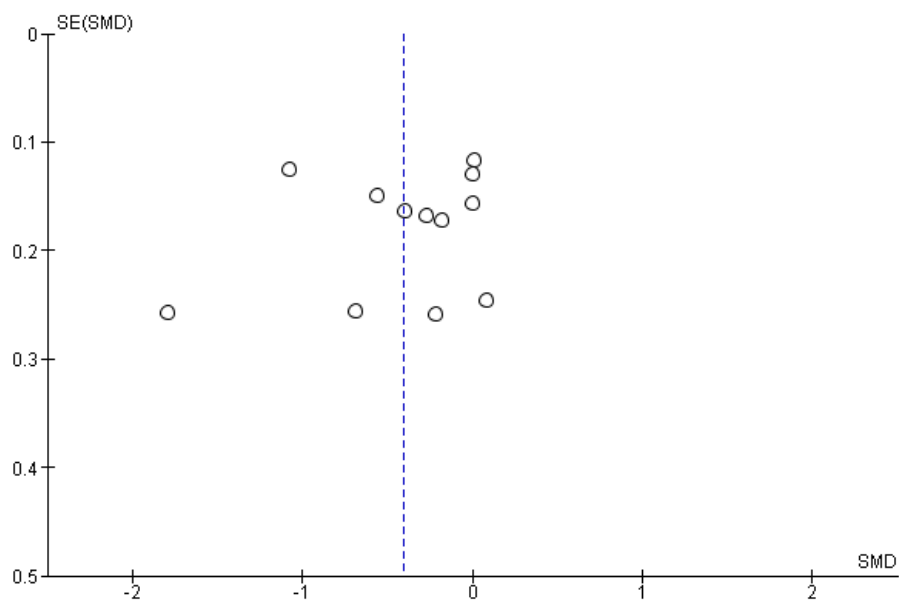

Funnel plot of Overall Studies

Supplement: Supplementary Material 2. [file epih-43-e2021090-suppl2.pdf]
