# Supplementary material for: The effect of peer support in diabetes self-management education on glycemic control in patients with type 2 diabetes: a systematic review and meta-analysis
Source: Epidemiol Health. 2021 Oct 22;43:e2021090. doi: 10.4178/epih.e2021090 (PMC8920738; doi:10.4178/epih.e2021090)
Supplement: Supplementary Material 3. [file epih-43-e2021090-suppl3.pdf]

Supplementary Material 3

Forest Plot of Subgroup Analysis

Study or Subgroup	Peer DSME Mean	Peer DSME SD	Peer DSME Total	Non-Peer DSME Mean	Non-Peer DSME SD	Non-Peer DSME Total	Weight	Std. Mean Difference IV, Random, 95% CI	Year
**1.3.1 Sample size <100**									
Samuel-Hodge 2009	7.4	0.1	101	7.4	0.1	69	8.7%	0.00 [-0.31, 0.31]	2009
Sinclair 2012	8.2	1.1	34	9.4	2.2	31	7.4%	-0.69 [-1.19, -0.19]	2012
Thanh 2013	7.11	0.49	42	7.94	0.43	44	7.4%	-1.79 [-2.29, -1.28]	2013
Siminerio 2013	8.63	1.2	31	8.89	1.2	29	7.4%	-0.21 [-0.72, 0.29]	2013
Tang 2015	8.3	2.4	34	8.1	2.2	32	7.5%	0.09 [-0.40, 0.57]	2015
Yin 2015	7.1	1.1	79	7.3	1.1	60	8.5%	-0.18 [-0.52, 0.16]	2015
Deng 2016	7.85	0.41	97	8.11	0.51	90	8.8%	-0.56 [-0.85, -0.27]	2016
Paz-Pacheco 2017	6.45	2.7	85	7.6	3.1	70	8.6%	-0.40 [-0.72, -0.08]	2017
**Subtotal (95% CI)**			**503**			**425**	**64.3%**	**-0.45 [-0.79, -0.11]**	
Heterogeneity: $Tau^2 = 0.20$; $Chi^2 = 44.20$, df = 7 (P < 0.00001); $I^2 = 84\%$									
Test for overall effect: Z = 2.59 (P = 0.009)									
**1.3.2 Sample size >=100**									
Lorig 2009	6.6	2.47	161	6.57	2.31	133	9.1%	0.01 [-0.22, 0.24]	2009
Less 2011	7.08	1.53	158	8.81	1.69	135	9.0%	-1.07 [-1.32, -0.83]	2011
Philis-Tsimikas 2011	9.1	2	64	9.7	2.3	81	8.6%	-0.27 [-0.60, 0.05]	2011
Riddell 2016	7.3	1.1	113	7.3	1.2	123	9.0%	0.00 [-0.26, 0.26]	2016
**Subtotal (95% CI)**			**496**			**472**	**35.7%**	**-0.33 [-0.87, 0.20]**	
Heterogeneity: $Tau^2 = 0.28$; $Chi^2 = 50.30$, df = 3 (P < 0.00001); $I^2 = 94\%$									
Test for overall effect: Z = 1.23 (P = 0.22)									
**Total (95% CI)**			**999**			**897**	**100.0%**	**-0.41 [-0.69, -0.13]**	
Heterogeneity: $Tau^2 = 0.21$; $Chi^2 = 94.87$, df = 11 (P < 0.00001); $I^2 = 88\%$									
Test for overall effect: Z = 2.89 (P = 0.004)									
Test for subgroup differences: $Chi^2 = 0.13$, df = 1 (P = 0.72), $I^2 = 0\%$									

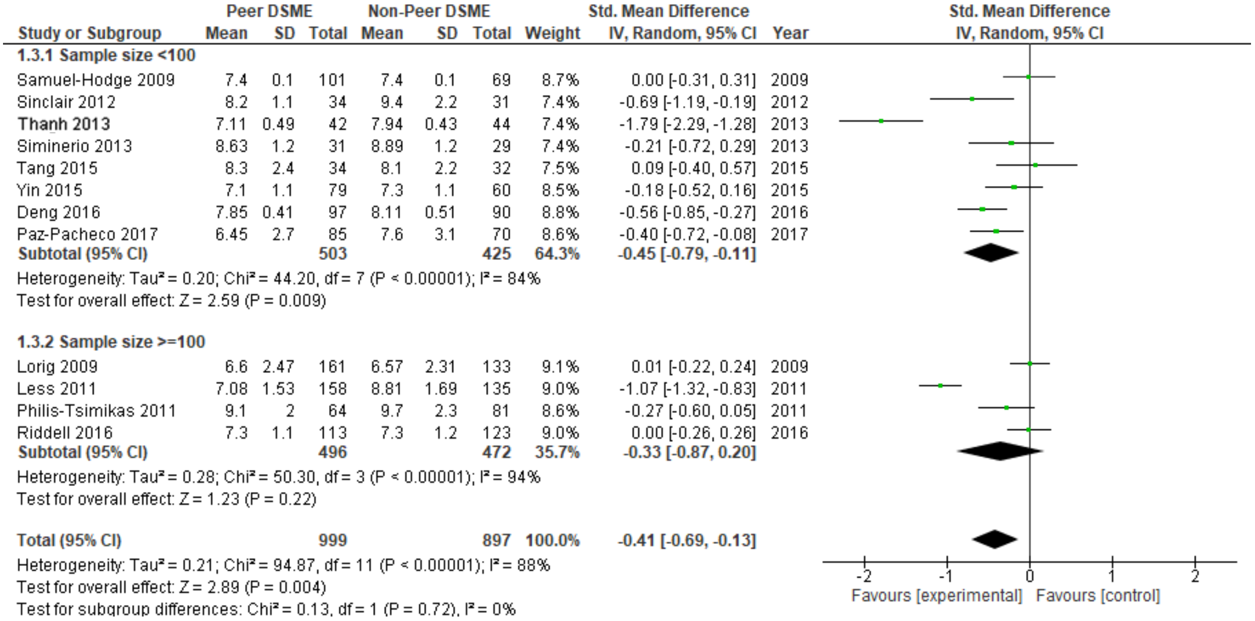

Fig 1. The effect Size and 95%CI of included studies based on sample size

Study or Subgroup	Peer DSME Mean	Peer DSME SD	Peer DSME Total	Non-Peer DSME Mean	Non-Peer DSME SD	Non-Peer DSME Total	Weight	Std. Mean Difference IV, Random, 95% CI	Year
**1.5.1 Duration of intervention <=6 months**									
Lorig 2009	6.6	2.47	161	6.57	2.31	133	9.1%	0.01 [-0.22, 0.24]	2009
Sinclair 2012	8.2	1.1	34	9.4	2.2	31	7.4%	-0.69 [-1.19, -0.19]	2012
Thanh 2013	7.11	0.49	42	7.94	0.43	44	7.4%	-1.79 [-2.29, -1.28]	2013
Siminerio 2013	8.63	1.2	31	8.89	1.2	29	7.4%	-0.21 [-0.72, 0.29]	2013
Yin 2015	7.1	1.1	79	7.3	1.1	60	8.5%	-0.18 [-0.52, 0.16]	2015
Paz-Pacheco 2017	6.45	2.7	85	7.6	3.1	70	8.6%	-0.40 [-0.72, -0.08]	2017
**Subtotal (95% CI)**			**432**			**367**	**48.4%**	**-0.52 [-0.96, -0.07]**	
Heterogeneity: $Tau^2 = 0.27$; $Chi^2 = 43.82$, df = 5 (P < 0.00001); $I^2 = 89\%$									
Test for overall effect: Z = 2.27 (P = 0.02)									
**1.5.2 Duration of intervention >6 months**									
Samuel-Hodge 2009	7.4	0.1	101	7.4	0.1	69	8.7%	0.00 [-0.31, 0.31]	2009
Philis-Tsimikas 2011	9.1	2	64	9.7	2.3	81	8.6%	-0.27 [-0.60, 0.05]	2011
Less 2011	7.08	1.53	158	8.81	1.69	135	9.0%	-1.07 [-1.32, -0.83]	2011
Tang 2015	8.3	2.4	34	8.1	2.2	32	7.5%	0.09 [-0.40, 0.57]	2015
Riddell 2016	7.85	0.41	97	8.11	0.51	90	8.8%	-0.56 [-0.85, -0.27]	2016
Deng 2016	7.3	1.1	113	7.3	1.2	123	9.0%	0.00 [-0.26, 0.26]	2016
**Subtotal (95% CI)**			**567**			**530**	**51.6%**	**-0.32 [-0.71, 0.08]**	
Heterogeneity: $Tau^2 = 0.22$; $Chi^2 = 50.51$, df = 5 (P < 0.00001); $I^2 = 90\%$									
Test for overall effect: Z = 1.57 (P = 0.12)									
**Total (95% CI)**			**999**			**897**	**100.0%**	**-0.41 [-0.69, -0.13]**	
Heterogeneity: $Tau^2 = 0.21$; $Chi^2 = 94.87$, df = 11 (P < 0.00001); $I^2 = 88\%$									
Test for overall effect: Z = 2.89 (P = 0.004)									
Test for subgroup differences: $Chi^2 = 0.44$, df = 1 (P = 0.51), $I^2 = 0\%$									

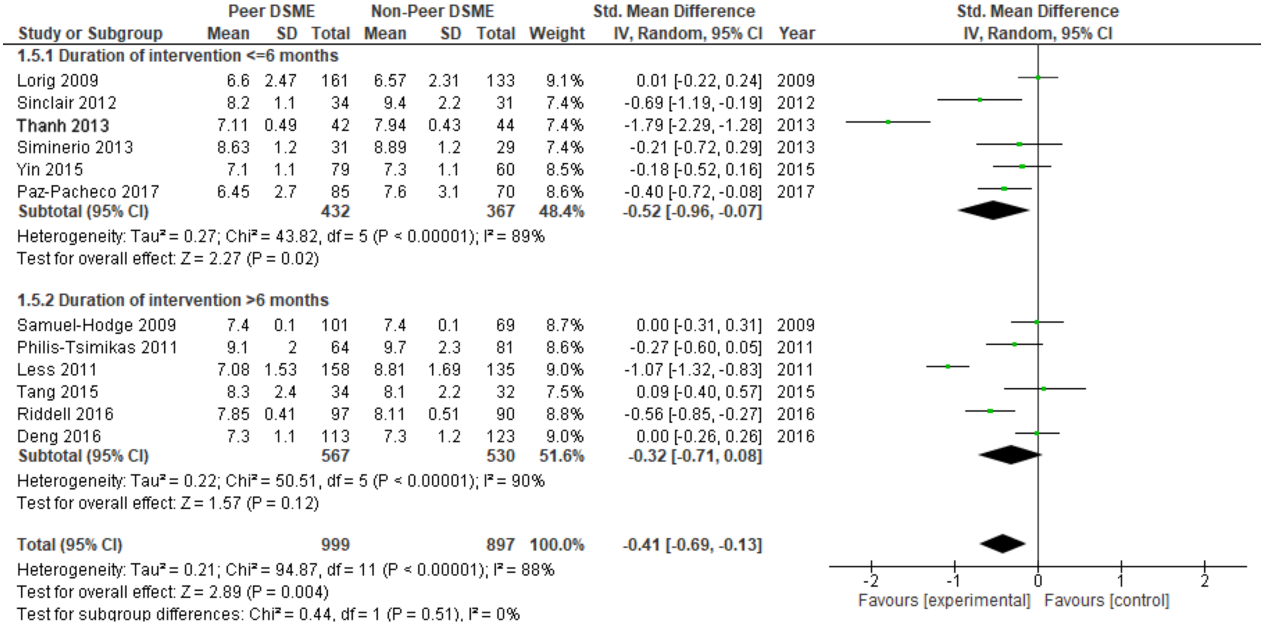

Fig 2. The effect Size and 95%CI of included studies based on duration of intervention

Study or Subgroup	Peer DSME Mean	Peer DSME SD	Peer DSME Total	Non-Peer DSME Mean	Non-Peer DSME SD	Non-Peer DSME Total	Weight	Std. Mean Difference IV, Random, 95% CI	Year
**1.5.1 HbA1c at baseline <8.5**									
Samuel-Hodge 2009	7.4	0.1	101	7.4	0.1	69	8.7%	0.00 [-0.31, 0.31]	2009
Lorig 2009	6.6	2.47	161	6.57	2.31	133	9.1%	0.01 [-0.22, 0.24]	2009
Less 2011	7.08	1.53	158	8.81	1.69	135	9.0%	-1.07 [-1.32, -0.83]	2011
Thanh 2013	7.11	0.49	42	7.94	0.43	44	7.4%	-1.79 [-2.29, -1.28]	2013
Yin 2015	7.1	1.1	79	7.3	1.1	60	8.5%	-0.18 [-0.52, 0.16]	2015
Tang 2015	8.3	2.4	34	8.1	2.2	32	7.5%	0.09 [-0.40, 0.57]	2015
Riddell 2016	7.3	1.1	113	7.3	1.2	123	9.0%	0.00 [-0.26, 0.26]	2016
Deng 2016	7.85	0.41	97	8.11	0.51	90	8.8%	-0.56 [-0.85, -0.27]	2016
Paz-Pacheco 2017	6.45	2.7	85	7.6	3.1	70	8.6%	-0.40 [-0.72, -0.08]	2017
**Subtotal (95% CI)**			**870**			**756**	**76.7%**	**-0.42 [-0.77, -0.07]**	
Heterogeneity: Tau^2 = 0.25; Chi^2 = 92.61, df = 8 (P < 0.00001); I^2 = 91%									
Test for overall effect: Z = 2.37 (P = 0.02)									
**1.5.2 Hba1c at baseline >=8.5**									
Philis-Tsimikas 2011	9.1	2	64	9.7	2.3	81	8.6%	-0.27 [-0.60, 0.05]	2011
Sinclair 2012	8.2	1.1	34	9.4	2.2	31	7.4%	-0.69 [-1.19, -0.19]	2012
Siminerio 2013	8.63	1.2	31	8.89	1.2	29	7.4%	-0.21 [-0.72, 0.29]	2013
**Subtotal (95% CI)**			**129**			**141**	**23.3%**	**-0.36 [-0.62, -0.10]**	
Heterogeneity: Tau^2 = 0.01; Chi^2 = 2.25, df = 2 (P = 0.32); I^2 = 11%									
Test for overall effect: Z = 2.72 (P = 0.006)									
**Total (95% CI)**			**999**			**897**	**100.0%**	**-0.41 [-0.69, -0.13]**	
Heterogeneity: Tau^2 = 0.21; Chi^2 = 94.87, df = 11 (P < 0.00001); I^2 = 88%									
Test for overall effect: Z = 2.89 (P = 0.004)									
Test for subgroup differences: Chi^2 = 0.07, df = 1 (P = 0.80), I^2 = 0%									

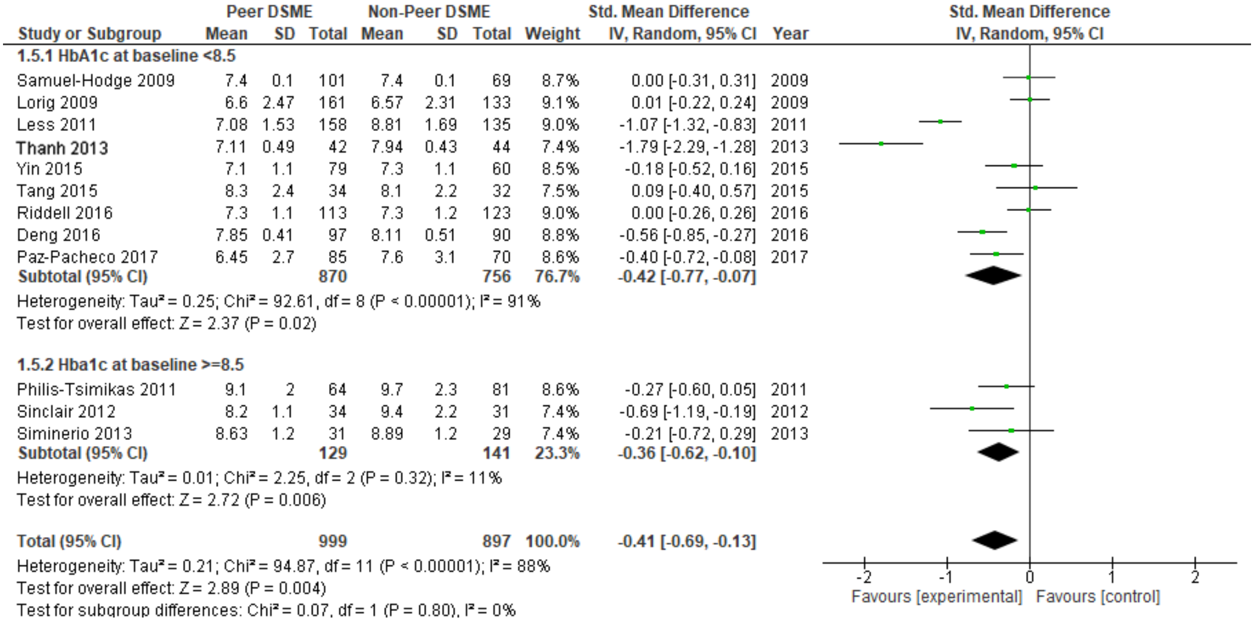

Fig 3. The effect Size and 95%CI of included studies based on the HbA1c baseline

Study or Subgroup	Peer DSME Mean	Peer DSME SD	Peer DSME Total	Non-Peer DSME Mean	Non-Peer DSME SD	Non-Peer DSME Total	Weight	Std. Mean Difference IV, Random, 95% CI	Year
**1.7.1 Individual**									
**Subtotal (95% CI)**			**0**			**0**		**Not estimable**	
Heterogeneity: Not applicable									
Test for overall effect: Not applicable									
**1.7.2 Group**									
Lorig 2009	6.6	2.47	161	6.57	2.31	133	9.1%	0.01 [-0.22, 0.24]	2009
Philis-Tsimikas 2011	9.1	2	64	9.7	2.3	81	8.6%	-0.27 [-0.60, 0.05]	2011
Sinclair 2012	8.2	1.1	34	9.4	2.2	31	7.4%	-0.69 [-1.19, -0.19]	2012
Deng 2016	7.85	0.41	97	8.11	0.51	90	8.8%	-0.56 [-0.85, -0.27]	2016
Riddell 2016	7.3	1.1	113	7.3	1.2	123	9.0%	0.00 [-0.26, 0.26]	2016
Paz-Pacheco 2017	6.45	2.7	85	7.6	3.1	70	8.6%	-0.40 [-0.72, -0.08]	2017
**Subtotal (95% CI)**			**554**			**528**	**51.5%**	**-0.28 [-0.51, -0.06]**	
Heterogeneity: Tau^2 = 0.05; Chi^2 = 16.70, df = 5 (P = 0.005); I^2 = 70%									
Test for overall effect: Z = 2.44 (P = 0.01)									
**1.7.3 Combination**									
Samuel-Hodge 2009	7.4	0.1	101	7.4	0.1	69	8.7%	0.00 [-0.31, 0.31]	2009
Less 2011	7.08	1.53	158	8.81	1.69	135	9.0%	-1.07 [-1.32, -0.83]	2011
Thanh 2013	7.11	0.49	42	7.94	0.43	44	7.4%	-1.79 [-2.29, -1.28]	2013
Siminerio 2013	8.63	1.2	31	8.89	1.2	29	7.4%	-0.21 [-0.72, 0.29]	2013
Yin 2015	7.1	1.1	79	7.3	1.1	60	8.5%	-0.18 [-0.52, 0.16]	2015
Tang 2015	8.3	2.4	34	8.1	2.2	32	7.5%	0.09 [-0.40, 0.57]	2015
**Subtotal (95% CI)**			**445**			**369**	**48.5%**	**-0.53 [-1.07, 0.02]**	
Heterogeneity: Tau^2 = 0.42; Chi^2 = 66.04, df = 5 (P < 0.00001); I^2 = 92%									
Test for overall effect: Z = 1.88 (P = 0.06)									
**Total (95% CI)**			**999**			**897**	**100.0%**	**-0.41 [-0.69, -0.13]**	
Heterogeneity: Tau^2 = 0.21; Chi^2 = 94.87, df = 11 (P < 0.00001); I^2 = 88%									
Test for overall effect: Z = 2.89 (P = 0.004)									
Test for subgroup differences: Chi^2 = 0.64, df = 1 (P = 0.42), I^2 = 0%									

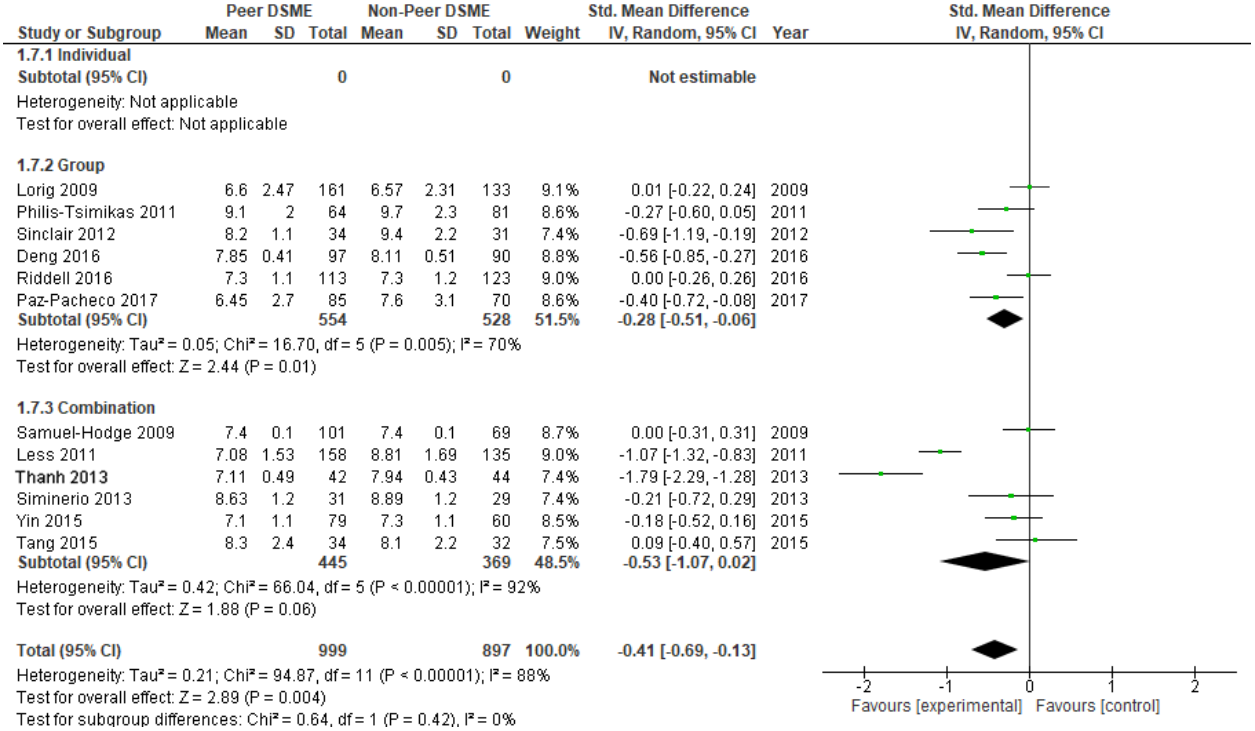

Fig 4. The effect Size and 95%CI of included studies based on type of delivery

Study or Subgroup	Peer DSME Mean	Peer DSME SD	Peer DSME Total	Non-Peer DSME Mean	Non-Peer DSME SD	Non-Peer DSME Total	Weight	Std. Mean Difference IV, Random, 95% CI	Year
**1.6.1 Low frequency of contact**									
Lorig 2009	6.6	2.47	161	6.57	2.31	133	9.1%	0.01 [-0.22, 0.24]	2009
Less 2011	7.08	1.53	158	8.81	1.69	135	9.0%	-1.07 [-1.32, -0.83]	2011
**Subtotal (95% CI)**			**319**			**268**	**18.2%**	**-0.53 [-1.60, 0.54]**	
Heterogeneity: Tau² = 0.58; Chi² = 40.11, df = 1 (P < 0.00001); I² = 98%									
Test for overall effect: Z = 0.98 (P = 0.33)									
**1.6.2 Moderate frequency of contact**									
**Thanh 2013**	7.11	0.49	42	7.94	0.43	44	7.4%	-1.79 [-2.29, -1.28]	2013
Siminerio 2013	8.63	1.2	31	8.89	1.2	29	7.4%	-0.21 [-0.72, 0.29]	2013
Riddell 2016	7.3	1.1	113	7.3	1.2	123	9.0%	0.00 [-0.26, 0.26]	2016
**Subtotal (95% CI)**			**186**			**196**	**23.7%**	**-0.65 [-1.71, 0.40]**	
Heterogeneity: Tau² = 0.82; Chi² = 38.78, df = 2 (P < 0.00001); I² = 95%									
Test for overall effect: Z = 1.22 (P = 0.22)									
**1.6.3 High frequency of contact**									
Samuel-Hodge 2009	7.4	0.1	101	7.4	0.1	69	8.7%	0.00 [-0.31, 0.31]	2009
Philis-Tsimikas 2011	9.1	2	64	9.7	2.3	81	8.6%	-0.27 [-0.60, 0.05]	2011
Sinclair 2012	8.2	1.1	34	9.4	2.2	31	7.4%	-0.69 [-1.19, -0.19]	2012
Tang 2015	8.3	2.4	34	8.1	2.2	32	7.5%	0.09 [-0.40, 0.57]	2015
Yin 2015	7.1	1.1	79	7.3	1.1	60	8.5%	-0.18 [-0.52, 0.16]	2015
Deng 2016	7.85	0.41	97	8.11	0.51	90	8.8%	-0.56 [-0.85, -0.27]	2016
Paz-Pacheco 2017	6.45	2.7	85	7.6	3.1	70	8.6%	-0.40 [-0.72, -0.08]	2017
**Subtotal (95% CI)**			**494**			**433**	**58.1%**	**-0.29 [-0.48, -0.10]**	
Heterogeneity: Tau² = 0.03; Chi² = 12.39, df = 6 (P = 0.05); I² = 52%									
Test for overall effect: Z = 2.93 (P = 0.003)									
**Total (95% CI)**			**999**			**897**	**100.0%**	**-0.41 [-0.69, -0.13]**	
Heterogeneity: Tau² = 0.21; Chi² = 94.87, df = 11 (P < 0.00001); I² = 88%									
Test for overall effect: Z = 2.89 (P = 0.004)									
Test for subgroup differences: Chi² = 0.63, df = 2 (P = 0.73), I² = 0%									

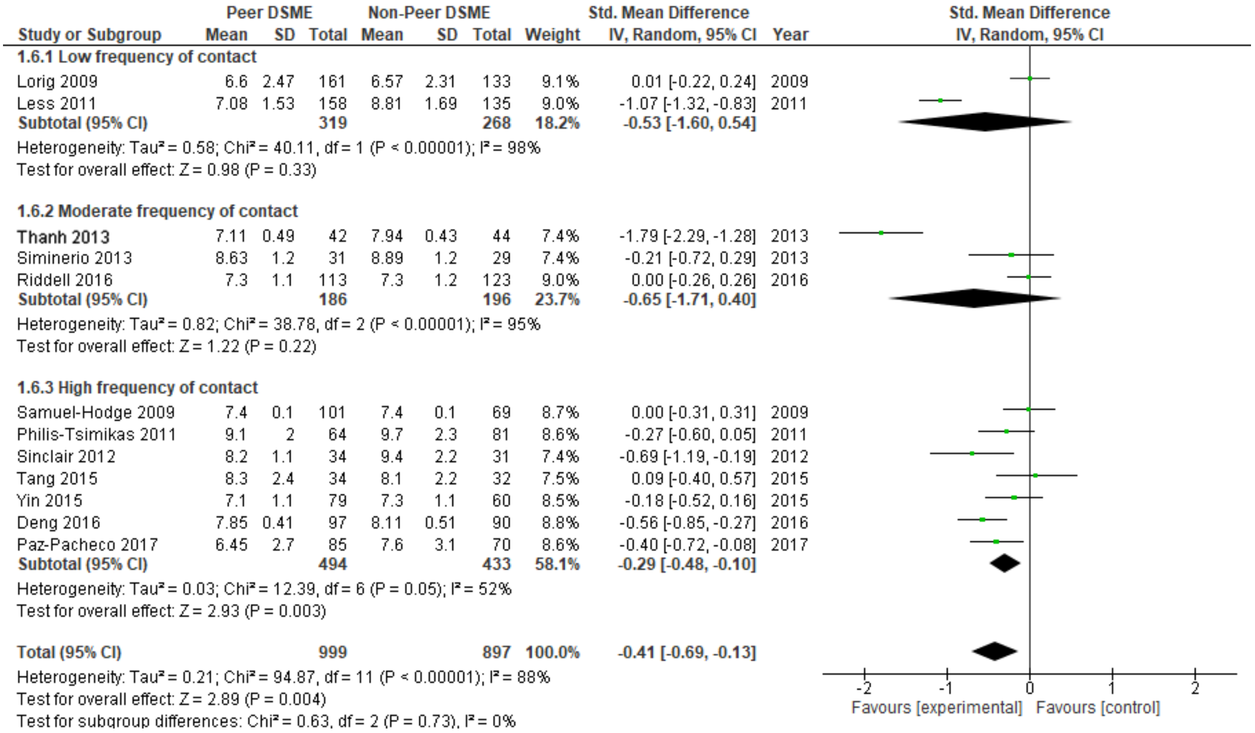

Fig. 5 The effect Size and 95%CI of included studies based on frequency of contact
